# Defecation pain and coccydynia due to an anteverted coccyx: a case report

**DOI:** 10.1186/1752-1947-6-175

**Published:** 2012-07-02

**Authors:** Omer Salar, Fizza Mushtaq, Mushtaq Ahmed

**Affiliations:** 1 Derby City General Hospital, Uttoxeter Road, Derby, DE22 3NE, UK; 2 Birmingham City Hospital, Dudley Road, Birmingham, B18 7QH, UK; 3 Russells Hall Hospital, Dudley, DY1 2HQ, UK

## Abstract

****Introduction**:**

Defecation pain is a common problem with many etiologies implicated. Elucidating a cause requires a thorough medical history, examination and appropriate investigations, which may include endoscopy, barium enema, examination under anesthesia and magnetic resonance imaging or computed tomography. Coccydynia is a term used to describe pain in the region of the coccyx, often due to abnormal mobility of the coccyx. Non-surgical management options remain the gold-standard for coccydynia with surgery being reserved for complicated cases.

****Case presentation**:**

This is a case of a 67-year-old Caucasian man who presented with a two-and-a-half-year history of worsening rectal pain.

****Conclusion**:**

To the best of our knowledge, we describe the first case in the literature of an abnormally mobile anteverted coccyx causing predominantly defecation pain and coccydynia, successfully treated by coccygectomy. When first-line investigations fail to elucidate a cause of defecation pain one must, in the presence of unusual symptoms, consider musculoskeletal pathologies emanating from the coccyx and an orthopedic consultation must then be sought for diagnostic purposes.

## **Introduction**

Defecation pain is a common problem with many causes implicated. Common causes include infective, neoplastic and anatomical or structural disorders. Coccydynia is a term used to describe pain in the region of the coccyx. Most cases have been demonstrated to be due to the abnormal mobility of the coccyx leading to fibrotic degeneration of the structure [1]. To the best of our knowledge, this is the first case report documenting an anteverted coccyx causing both chronic defecation pain and coccydynia.

## **Case presentation**

A 67-year-old man presented with a two-and-a-half-year history of worsening rectal pain developing an hour before defecation and lasting for several hours afterwards. Otherwise our patient had normal bowel habits. There was some pain when sitting on a hard surface that lessened when sitting on a soft cushion. No pain was experienced on walking or standing. Of note, our patient had type II diabetes and ischemic heart disease. He denied previous musculoskeletal problems, including back pain.

The rectal pain was thoroughly investigated by a consultant colorectal surgeon. Rigid sigmoidoscope and endoanal ultrasound investigations were normal. A barium enema revealed mild diverticular disease and an anorectal examination under anesthesia and subsequent biopsy revealed only a benign polyp. Double contrast magnetic resonance imaging (MRI) of his pelvis revealed no soft tissue abnormality.

**Figure 1:**
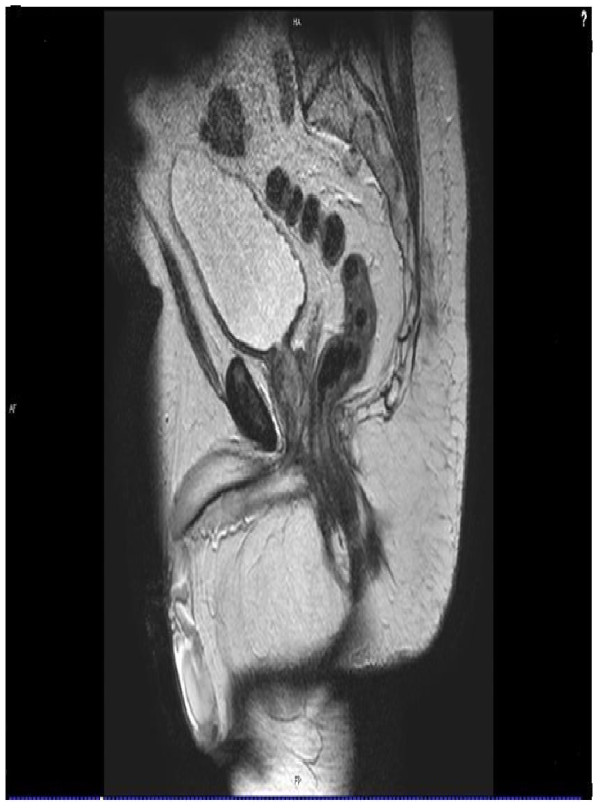
T2-weighted magnetic resonance image showing an anteverted coccyx and rectal impingement.

**Figure 2:**
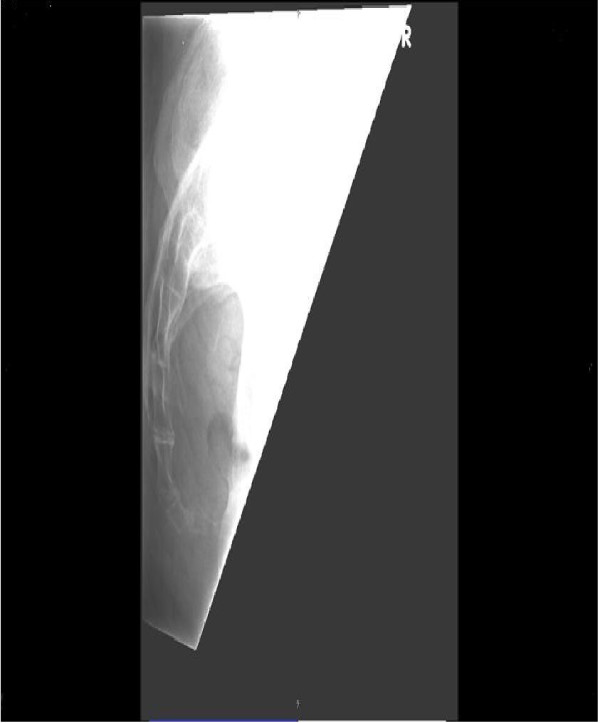
Lateral view X-ray showing an anteverted coccyx.

Our patient was subsequently referred for consultation with an orthopedic surgeon. A physical examination revealed a patient of medium build with palpation tenderness over the tip of his coccyx, which was significantly anteverted and mobile. No tenderness was elicited over the sacroiliac joint or lumbar spine. A straight leg raise to 90 degrees was achieved and there was no neurovascular deficit in his limbs. A review of the MRI scans of his pelvis (Figures 1 and 2) and plain X-rays (Figure 2) confirmed the diagnosis of an elongated, anteverted coccyx protruding into the rectum. A standard coccygectomy was carried out, after which our patient’s symptoms settled and he was discharged two months after the surgery with a good outcome.

## **Discussion**

Defecation pain is a symptom known to be caused by chronic coccydynia; especially before defecating or when constipated. Pain on defecation is a common presenting complaint. Nathan *et al*. describe the various causes of coccydynia and divide the etiology of coccydynia into three main categories [2]. Coccydynia can be idiopathic or traumatic in nature; various pathologies, such as those listed below, can be causative factors. Neoplastic pathologies include rectal adenocarcinomas, squamous cell anal carcinomas and rectal lymphomas being the most common. Infective processes include abscesses and anal fistulas, diverticulitis and anal papillitis and cryptitis. The final subcategory of causes of coccydynia includes somatic disorders.

The coccyx consists of three to five vertebral units that, except for the first, are fused together. Anatomically, the ventral surface is concave with grooves indicating lines of fusion. The dorsal aspect is convex and displays similar lines of fusion as well as multiple paired tubercles known as coccygeal articular processes [1,3-5].

Postacchini and Massobrio described four types of configurations of the coccyx and named them type I to type IV [3,4]. In type I, the coccyx is curved slightly forward, apex facing inferiorly and caudally. In type II, there is a more notable forward curvature and the apex extends forwards. In type III, the coccyx sharply curves to the ventral side. Finally, in type IV, anatomically there is joint subluxation at either the sacrococcygeal or intercoccygeal joint [3]. The majority of coccydynia occurs in conjunction with either a subluxed or hypermobile coccyx and this pathological instability has for some time been implicated as a cause of chronic inflammatory changes [1,3-6].

Non-surgical managements have remained the gold standard for treatment of coccydynia, such as seat cushioning, coccygeal massage, stretching and manipulation, local injection of steroids or pain controlling anesthetics [3,7]. Conservative measures provide symptomatic relief in approximately 90 % of simple cases [8,9]. Surgical intervention is typically reserved for patients with evidence of coccygeal instability, as described by Postacchini and Massobrio, such as subluxation and hypermobility or spicule formation with or without chronic symptoms that do not respond to non-surgical measures [1,10-15].

Long-term evidence exists for coccygectomy as a treatment for intractable coccydynia. Several investigators have reported results ranging from 60 % to 92 % success [10-15] whereas other authors advise against surgery. In a recent case series, 71 % of patients benefited from coccygectomy and, in particular, the authors noted a correlation between histological findings and perceived benefit [1].

## **Conclusion**

Investigating a patient with defecation pain requires a careful history and examination, including digital rectal examination. X-rays and endoscopy would be the first steps in determining a diagnosis. Subsequent investigations may include anorectal examination under anesthesia and barium enemas, progressing to MRI of the pelvis, particularly in the case of recurrent complicated infective processes. When first-line investigations fail to elucidate a cause of defecation pain, one must, in the presence of unusual symptoms, consider musculoskeletal pathologies emanating from the coccyx and an orthopedic consultation must then be sought for diagnostic purposes.

## **Consent**

Written informed consent was obtained from the patient for publication of this case report and any accompanying images. A copy of the written consent is available for review by the Editor-in-Chief of this journal.

## **Competing interests**

The authors declare that they have no competing interests.

## **Authors’ contributions**

OS made significant contributions to the design, conception, writing, drafting and final submission of the manuscript. FM contributed to the design, drafting and final editing of the manuscript. MA was the surgeon and overall supervisor for this case report; conceptualized the manuscript and approved the final draft. All authors read and approved the final manuscript.
